# Supplementary material for: Serotonergic receptor binding in the brainstem in the Sudden Infant Death Syndrome in a high-risk population
Source: PLoS One. 2025 Sep 10;20(9):e0330940. doi: 10.1371/journal.pone.0330940 (PMC12422518; doi:10.1371/journal.pone.0330940)
Supplement: S1 File — Supplemental Tables 1–5 provide additional information regarding alcohol and smoking exposure in different cohorts examined in the study. Supplemental Table 6 provides 3H-8-OH-DPAT binding data in SIDS vs all KCOD controls (pre- and postdischarge). Supplemental Table 7 provides 3H-8-OH-DPAT binding data in the South African cohort only. Supplemental Table 8 provides cohort information across tables and figures. (PDF) [file pone.0330940.s001.pdf]

Supplemental 1

Kinney et al. Serotonergic Receptor Binding in the Brainstem in the Sudden Infant Death Syndrome in a High-Risk Population

Supplemental 1_Table 1. Exposure information through pregnancy and by trimesters: Combined South Africa and Northern Plains cohorts.

	n	Mean	STD	Median	Min	Max	p-value
**Exposure through pregnancy**							
**N Drinks in Pregnancy**							0.699
SIDS	14	25.8	54.9	8.8	0	210.8	
PostKCOD	10	25.3	35.4	8.3	0	102.5	
**Average Cigarettes per week**							1.000
SIDS	13	24.6	19.8	20.2	0.1	62.3	
PostKCOD	8	26.8	23.9	28.7	0	58.6	
**Drinks by Trimester**							
**Trimester 1**							0.276
SIDS	14	7.6	21.1	0	0	80.1	
PostKCOD	10	10.1	13.6	3.6	0	37.5	
**Trimester 2**							0.441
SIDS	14	14.5	25.6	2.1	0	94.8	
PostKCOD	10	13.5	28.3	0	0	69.2	
**Trimester 3**							0.622
SIDS	14	3.7	9.6	0	0	35.9	
PostKCOD	10	1.6	4.6	0	0	14.5	
**Average Cigarettes per Week by Trimester**							
**Trimester 1**							0.720
SIDS	13	24.1	19.8	21.2	0	61.7	
PostKCOD	8	19.2	20.3	12.1	0	52.4	
**Trimester 2**							0.914
SIDS	13	25.0	21.3	21.5	0	64.4	
PostKCOD	8	27.7	23.8	27.6	0	60.0	
**Trimester 3**							0.755
SIDS	13	24.7	19.1	21.3	0.2	60.4	
PostKCOD	7	27.7	31.8	7.7	0	67.6	

Legend. Abbreviations. n=number; N=number; STD, standard deviation

Supplemental 1_Table 2. Exploratory analysis of alcohol and tobacco in SIDS by preterm vs. term. Combined South Africa and Northern Plains cohorts.

**SIDS ONLY**	n	Mean	STD	Median	Min	Max	p-value
**N Drinks in Pregnancy**							0.604
Preterm	7	39.3	76.49	12.3	0.0	210.7	
Term	7	12.3	15.71	0.0	0.0	35.1	
**Average Cigarettes per week**							0.371
Preterm	6	30.6	15.92	25.1	17.1	57.8	
Term	7	19.4	22.49	19.1	0.1	62.3	
**Drinks by Trimester**							
**Trimester 1**							0.160
Preterm	7	14.2	29.24	5.2	0.0	80.1	
Term	7	1.0	2.64	0.0	0.0	7.0	
**Trimester 2**							0.689
Preterm	7	19.4	34.68	4.2	0.0	94.8	
Term	7	9.6	12.57	0.0	0.0	26.6	
**Trimester 3**							0.937
Preterm	7	5.6	13.40	0.0	0.0	35.9	
Term	7	1.7	3.50	0.0	0.0	9.3	
**Average Cigarettes per Week by Trimester**							
**Trimester 1**							0.245
Preterm	6	29.5	15.03	24.9	12.2	54.5	
Term	7	19.5	23.25	17.6	0.0	61.7	
**Trimester 2**							0.447
Preterm	6	30.3	18.30	26.2	12.6	60.6	
Term	7	20.4	23.98	18.3	0.0	64.4	
**Trimester 3**							0.200
Preterm	6	32.1	15.23	29.9	16.3	58.8	
Term	7	18.3	20.78	17.2	0.2	60.4	

	**Preterm [n (%)]**	**Term [n (%)]**	**P-value**
Any Alcohol	5 (71)	3 (43)	0.5921

Legend. Abbreviations. n=number; N=number; STD, standard deviation

Supplemental 1_Table 3. Exploratory analysis of alcohol and tobacco in Post KCOD by preterm vs. term. Combined South Africa and Northern Plains cohorts.

KCOD ONLY	n	Mean	STD	Median	Min	Max	Wilcoxon p-value
**N Drinks in Pregnancy**							0.6823
Preterm	5	34.9	49.03	0.0	0.0	102.5	
Term	5	15.6	13.59	12.3	1.6	29.9	
**Average Cigarettes per week**							0.4939
Preterm	4	31.4	24.65	31.2	4.6	58.6	
Term	4	22.2	25.89	19.9	0.0	49.1	
**N Drinks by Trimester**							
**Trimester 1**							0.3575
Preterm	5	8.1	16.48	0.0	0.0	37.5	
Term	5	12.2	11.48	11.3	0.0	29.7	
**Trimester 2**							0.4604
Preterm	5	26.9	36.80	0.0	0.0	69.2	
Term	5	0.2	0.45	0.0	0.0	1.0	
**Trimester 3**							0.2126
Preterm	5	0.0	0.00	0.0	0.0	0.0	
Term	5	3.2	6.34	0.0	0.0	14.5	
**Average Cigarettes per Week by Trimester**							
**Trimester 1**							0.3460
Preterm	4	26.5	20.30	24.8	4.3	52.4	
Term	4	11.9	20.18	2.8	0.0	41.9	
**Trimester 2**							0.6762
Preterm	4	30.6	21.86	27.6	7.2	60.0	
Term	4	24.7	28.65	23.2	0.0	52.5	
**Trimester 3**							0.8643
Preterm	3	24.5	33.73	7.7	2.4	63.3	
Term	4	30.1	35.28	26.4	0.0	67.6	

	**Preterm [n (%)]**	**Term [n (%)]**	**P-value**
Any Alcohol	2 (40)	5 (100)	0.1667

Legend. Abbreviations. n=number; N=number; STD, standard deviation

Supplemental 1_Table 4. Exploratory analysis of alcohol and tobacco in preterm infants by diagnosis: Combined South Africa and Northern Plains cohorts.

**Preterms ONLY**	n	Mean	STD	Median	Min	Max	p-value
**Drinks in Pregnancy**							0.7426
SIDS	7	39.3	76.49	12.3	0.0	210.7	
Other Known Cause	5	34.9	49.03	0.0	0.0	102.5	
**Average Cigarettes per week**							1.0000
SIDS	6	30.6	15.92	25.1	17.1	57.8	
Other Known Cause	4	31.4	24.65	31.2	4.6	58.6	
**N Drinks by Trimester**							
**Trimester 1**							0.5563
SIDS	7	14.2	29.24	5.2	0.0	80.1	
Other Known Cause	5	8.1	16.48	0.0	0.0	37.5	
**Trimester 2**							0.9325
SIDS	7	19.4	34.68	4.2	0.0	94.8	
Other Known Cause	5	26.9	36.80	0.0	0.0	69.2	
**Trimester 3**							0.2851
SIDS	7	5.6	13.40	0.0	0.0	35.9	
Other Known Cause	5	0.0	0.00	0.0	0.0	0.0	
**Average Cigarettes per Week by Trimester**							
**Trimester 1**							0.7564
SIDS	6	29.5	15.03	24.9	12.2	54.5	
Other Known Cause	4	26.5	20.30	24.8	4.3	52.4	
**Trimester 2**							0.9174
SIDS	6	30.3	18.30	26.2	12.6	60.6	
Other Known Cause	4	30.6	21.86	27.6	7.2	60.0	
**Trimester 3**							0.5367
SIDS	6	32.1	15.23	29.9	16.3	58.8	
Other Known Cause	3	24.5	33.73	7.7	2.4	63.3	

	**SIDS [n (%)]**	**KCOD [n (%)]**	**P-value**
Any Alcohol	5 (71)	2 (40)	0.5581

Legend. Abbreviations. n=number; N=number; STD, standard deviation

Supplemental 1_Table 5. Exploratory analysis of alcohol and tobacco in term infants by diagnosis: Combined South Africa and Northern Plains cohorts.

**Terms ONLY**	n	Mean	STD	Median	Min	Max	p-value
N Drinks in Pregnancy							0.3426
SIDS	7	12.3	15.71	0.0	0.0	35.1	
Other Known Cause	5	15.6	13.59	12.3	1.6	29.9	
Average Cigarettes per week							0.9266
SIDS	7	19.4	22.49	19.1	0.1	62.3	
Other Known Cause	4	22.2	25.89	19.9	0.0	49.1	
N Drinks by Trimester							p-value
**Trimester 1**							*0.0525*
SIDS	7	1.0	2.64	0.0	0.0	7.0	
Other Known Cause	5	12.2	11.48	11.3	0.0	29.7	
**Trimester 2**							0.3547
SIDS	7	9.6	12.57	0.0	0.0	26.6	
Other Known Cause	5	0.2	0.45	0.0	0.0	1.0	
**Trimester 3**							0.7773
SIDS	7	1.7	3.50	0.0	0.0	9.3	
Other Known Cause	5	3.2	6.34	0.0	0.0	14.5	
Average Cigarettes per Week by Trimester	n	Mean	STD	Median	Min	Max	p-value
**Trimester 1**							0.3460
SIDS	7	19.5	23.25	17.6	0.0	61.7	
Other Known Cause	4	11.9	20.18	2.8	0.0	41.9	
**Trimester 2**							0.6762
SIDS	7	20.4	23.98	18.3	0.0	64.4	
Other Known Cause	4	24.7	28.65	23.2	0.0	52.5	
**Trimester 3**							0.8643
SIDS	7	18.3	20.78	17.2	0.2	60.4	
Other Known Cause	4	30.1	35.28	26.4	0.0	67.6	

	**SIDS [n (%)]**	**KCOD [n (%)]**	**P-value**
Any Alcohol	3 (43)	5 (100)	*0.0808*

Legend. Abbreviations. n=number; N=number; STD, standard deviation

Supplemental 1_Table 6. SIDS vs. all KCOD controls (pre- and postdischarge): Effect of diagnosis controlling for PCA on ^{3}H-8-OH-DPAT binding in the brainstem: Combined South Africa and Northern Plains cohorts.

	Diagnosis					PCA		Dx by PCA Interaction
	SIDS		Other Known Cause		p-value	Beta	p value	p-value
	n	Mean ± SE	n	Mean ± SE				
**Mid Medulla**								
RO/RMg	12	32.9 ± 4.7	19	30.4 ± 3.6	0.690	-0.44 (0.25)	*0.090*	0.380
HG	13	11.4 ± 1.0	20	7.4 ± 0.8	**0.004**	-0.24 (0.05)	**<0.001**	0.140
DMX	12	12.5 ± 1.4	20	9.2 ± 1.0	*0.080*	-0.17 (0.07)	**0.030**	0.280
NTS	13		20		*0.050*			**0.010**
S5	13	28.2 ± 3.4	20	21.1 ± 2.6	0.120	-0.3 (0.19)	0.120	0.140
Centralis	13		20		0.110			**0.030**
ARC	12	4.4 ± 0.9	19	4.0 ± 0.6	0.740	-0.03 (0.04)	0.450	0.150
PIO	13		20		0.350			**0.030**
MAO	13		20		0.630			**0.040**
**Rostral Medulla**								
RO/RMg	14	46.2 ± 6.	20	47.2 ± 5.2	0.910	-1.14 (0.36)	**0.004**	0.520
GC	14	26.5 ± 3.0	20	23.2 ± 2.5	0.420	-0.71 (0.17)	**<0.001**	0.507
PGCL	14	27.7 ± 3.4	20	24.2 ± 2.8	0.450	-0.75 (0.19)	**<0.001**	0.460
IRZ	14	26.6 ± 3.0	20	23.4 ± 2.4	0.420	-0.72 (0.17)	**<0.001**	0.530
S5	14	24.4 ± 3.0	20	19.8 ± 2.4	0.260	-0.63 (0.17)	**<0.001**	0.400
ARC	14	6.8 ± 0.9	19	5.5 ± 0.8	0.310	-0.12 (0.05)	**0.030**	0.080
PIO	14	4.6 ± 0.7	20	3.5 ± 0.6	0.260	-0.07 (0.04)	*0.090*	*0.090*
DAO	14	21.2 ± 2.8	19	20.0 ± 2.4	0.760	-0.56 (0.16)	**0.002**	0.910
**Rostral Pons**								
MR	8	45.4 ± 12.0	13	41.1 ± 9.2	0.790	-0.04 (0.59)	0.940	0.550
LC	7	7.3 ± 2.7	12	16.2 ± 2.0	**0.030**	-0.04 (0.13)	0.760	0.800
PO	8	11.4 ± 4.5	13	22.2 ± 3.4	*0.080*	-0.04 (0.22)	0.850	0.360
DR	6	51.8 ± 13.7	12	54.7 ± 9.3	0.870	0.51 (0.6)	0.410	0.770
BP	9	5.4 ± 2.0	13	6.0± 1.6	0.800	-0.11 (0.1)	0.270	0.660

Legend. Significant p-values (<0.05) are bold. Marginal p-values (<0.1) are in italics. Abbreviations: PCA, postconceptional age; Ave, average; SIDS, sudden infant death syndrome; HG, hypoglossal nucleus; DMX, dorsal motor nucleus of the vagus; NTS, nucleus of the solitary tract; S5, spinal trigeminal nucleus; CEN, centralis; PIO, principal inferior olive; MAO, medial accessory olive; ARC, arcuate nucleus, RO/RMg, raphe obscurus/Raphe Magnus; GC, gigantocellularis; PGCL, paragigantocellularis lateralis; DAO, dorsal accessory olive; MR, median raphe; LC, locus coeruleus; PO, nucleus pontis oralis; BP, basis pontis.

Supplementary 1_ Table 7. 5-HT$_{1A}$ binding in brainstem nuclei in Cape Coloured in South Africa only.

	Diagnosis					PCA		**Dx by PCA Interaction**
	SIDS		Other Known Cause		p-value	Beta	p-value	p-value
	n	Mean ± SE	n	Mean ± SE				
**Mid Medulla**								
RO/RMg	11	31.1 ± 4.8	7	27.3 ± 6.3	0.646	-0.71	*0.073*	0.908
HG	12	10.8 ± 1.0	8	6.5 ± 1.2	**0.014**	-0.26	**0.003**	0.237
DMX	11	12.3 ± 1.4	8	8.2 ± 1.7	*0.081*	-0.19	*0.084*	0.492
NTS	12	16.2 ± 1.7	8	8.1 ± 2.2	**0.010**	-0.53	**0.001**	*0.060*
S5	12	27.1 ± 3.4	8	22.1 ± 4.3	0.387	-0.55	*0.055*	0.486
Centralis	12	20.0 ± 1.9	8	14.6 ± 2.4	0.103	-0.72	**0.001**	0.436
ARC	11	4.2 ± 0.9	7	3.3 ± 1.1	0.499	-0.12	*0.086*	0.965
PIO	12	4.0 ± 0.7	8	2.3 ± 0.9	0.157	-0.13	**0.032**	0.395
MAO	12	20.7 ± 3.1	8	11.8 ± 3.9	*0.092*	-0.88	**0.002**	0.331
**Rostral Medulla**								
RO/RMg	13	41.5 ± 5.5	8	38.2 ± 7.23	0.7209	-1.34	**0.0066**	0.7881
GC	13	23.3 ± 2.9	8	19.5 ± 3.79	0.4371	-0.91	**0.0009**	0.6082
PGCL	13	24.1 ± 2.8	8	18.1 ± 3.77	0.2264	-0.97	**0.0005**	0.9389
IRZ	13	23.2 ± 2.7	8	20.0 ± 3.60	0.4921	-0.95	**0.0004**	0.5542
S5	13	21.4 ± 2.9	8	15.9 ± 3.76	0.2636	-0.81	**0.0022**	0.8826
ARC	13	6.5 ± 0.9	7	5.3 ± 1.28	0.4504	-0.16	**0.0436**	0.1786
PIO	13	4.3 ± 0.7	8	2.8 ± 0.93	0.2245	-0.12	**0.0436**	0.2259
DAO	13	18.3 ± 2.4	8	17.6 ± 3.22	0.8625	-0.66	**0.0033**	0.8346
**Rostral Pons**								
MR	7	49.2 ± 14.4	3	26.5 ± 21.9	0.413	-0.34	0.743	0.990
LC	6	7.3 ± 2.1	3	10.9 ± 2.9	0.342	-0.04	0.803	0.570
PO	7	11.7 ± 3.2	3	10.5 ± 4.9	0.841	-0.31	0.216	0.896
DR	5	59.8 ± 17.2	3	42.9 ± 21.2	0.562	0.65	0.566	0.738
BP	8	5.4 ± 2.1	3	6.8 ± 3.4	0.749	-0.15	0.353	0.951
	Means estimated for PCA = 48.8, Mean PCA							

Legend. There are a total of 21 subjects (SIDS n = 13) and PostKCOD (n = 8) from South Africa only, with a total of 24 subjects in the combined South Africa and Northern Plains group. We highlight the major findings. There is n = 1 fewer SIDS cases and n = 2 fewer PostKCOD cases in the South Africa group than the combined groups. Despite this small difference in sample size, the findings in binding are essentially the same. There are statistically significant differences in binding (mean or age vs. diagnosis) between the two groups in both the hypoglossal and nucleus of the solitary tract. In the South Africa group there is a marginal statistical difference in the medial accessory olive (p = 0.09) by diagnosis, in contrast to a significant difference (p = 0.03) (age vs. interaction) in the same nucleus when cases from South Africa and the Northern Plains are combined

Supplemental 1_Table 8. Cohort analyses across Tables and Figures.

	Demographics		5-HT_{1A} Analysis		Exposure Analysis		Maternal socioeconomic characteristics	
Combined South Africa and Northen Plains Cohort	+	Tables 1 and 2	+	Figures 1-4<br>Tables 5-7	+	Table 3<br>Table 8<br>S. Tables 1-5	-	
South Africa Cohort only	-		+	S. Tables 6 and 7	-		+	Table 4

The Table summarizes the use of the different sites (South Africa and Northern Plains) in data analyses across the Tables and Figures within the text. It is provided here as a reference to the reader.
