# Supplementary material for: Serotonergic receptor binding in the brainstem in the Sudden Infant Death Syndrome in a high-risk population
Source: PLoS One. 2025 Sep 10;20(9):e0330940. doi: 10.1371/journal.pone.0330940 (PMC12422518; doi:10.1371/journal.pone.0330940)
Supplement: Attachment — Submitted filename: Review SID.docx [file pone.0330940.s003.docx]

**Review for: “Serotonergic Receptor Binding in the Brainstem in the Sudden Infant Death Syndrome in a High-Risk Population”**

**Overall Recommendation: Major Revision**

This is an important and timely manuscript investigating the serotonergic underpinnings of Sudden Infant Death Syndrome (SIDS) in high-risk, socioeconomically disadvantaged populations. The use of the Safe Passage Study cohort provides an exceptional dataset, and the findings related to altered 5-HT1A binding—especially in premature SIDS cases—represent a potentially novel mechanism of pathogenesis. However, the manuscript would benefit from substantial revisions and additional clarifications in methodology, interpretation, and contextualization of the results.

**1. Title and Abstract**

**Comments:**

- The title is accurate and specific.
- The abstract concisely summarizes the study objectives and findings.

**Suggestions:**

- Include in the abstract a sentence highlighting the novel finding of increased 5-HT1A binding in premature SIDS infants.
- Clarify early that the study was unable to test the influence of maternal smoking/drinking due to high prevalence in both groups.

**2. Introduction**

**Comments:**

- The introduction is thorough and well-cited, providing a strong rationale for investigating serotonergic abnormalities in SIDS.
- Prior work is appropriately contextualized.

**Suggestions:**

- Consider emphasizing earlier into the introduction, that prior studies observed *reduced* 5-HT1A binding in SIDS to better contrast the present findings.
- Add a brief explanation (maybe a paragraph) of 5-HT1A receptor function in early brain development to frame the broader physiological implications.

**3. Materials and Methods**

**Study Design & Ethics:**

- The longitudinal design and ethical rigor are commendable.
- Tissue handling, recruitment strategy, and data integration are well described.

**Suggestions:**

- Provide a power analysis or justification for the sample size given the number of comparisons.
- Acknowledge the control group limitations more clearly (e.g., KCOD infants are not necessarily "healthy" controls).
- Clarify whether blinding was employed during receptor quantification.
- Consider discussing whether post-mortem interval was controlled beyond the statistical statement that it had no effect.

**Experimental Assays:**

- Autoradiography using 3H-8-OH-DPAT is an accepted method for 5-HT1A binding measurement.
- Imaging and densitometry are appropriate.

**Suggestions for Complementary Approaches:**

- Immunohistochemistry or in situ hybridization to support autoradiographic findings.
- Dual labeling with synaptic markers (e.g., PSD-95) or neuroinflammatory markers (e.g., Iba-1) may provide additional insight.
- Future studies should consider methylation analysis of the HTR1A gene in placental or brain tissue.

**4. Results**

**Findings:**

- Data clearly presented.
- The tables and figures are well designed.

**Key Result:**

- Elevated 5-HT1A binding in premature SIDS infants, particularly in the hypoglossal nucleus and NTS.

**Suggestions:**

- Provide effect sizes and confidence intervals for binding comparisons to emphasize magnitude.
- Consider modeling developmental trajectories of 5-HT1A binding using nonlinear regressions (e.g., splines).
- Immunohistochemistry or Western blotting for 5-HT1A expression would provide confirmatory spatial and expression-level data.
- Consider dual labeling with markers of apoptosis, microglial activation (Iba-1), or synaptic maturation (e.g., synaptophysin) to contextualize receptor changes.
- MRI-based volumetrics or Diffusion Tensor Imaging (DTI) in other studies may offer complementary non-destructive data in future cohorts.
- Highlight that inclusion of pre-discharge controls (PreKCOD) strengthens the interpretation of age-related trends.

**Missing Considerations:**

- Sex-specific analysis: Are there sex differences in receptor binding or SIDS incidence in this cohort?
- Cause-of-death heterogeneity in controls: Address whether the control group includes heterogeneous mechanisms of death that could confound comparisons.

**5. Discussion**

**Strengths:**

- Insightful and integrative, placing neurobiological data within a public health framework.
- The discussion of fetal programming, serotonopathy subtypes, and socioeconomic stressors is comprehensive.

**Suggestions:**

- Expand on potential mechanisms of elevated 5-HT1A binding (e.g., stress-induced overexpression, altered feedback signaling).
- Discuss possible functional implications: does increased 5-HT1A binding imply increased inhibitory tone in cardiorespiratory centers?
- Briefly explore how this subset of "preterm SIDS" may represent a distinct pathophysiological endotype.
- Consider referencing animal studies of maternal stress or early-life adversity affecting serotonergic signaling.

**6. Limitations**

**Comments:**

- The authors appropriately acknowledge key limitations, including sample size and exposure homogeneity.

**Suggestions:**

- Note the lack of external validation (e.g., protein expression or imaging).
- Discuss the potential influence of systemic infections in controls on receptor binding.

**7. Conclusions and Future Directions**

**Comments:**

- The call for further research and integration of social determinants of health is persuasive and appropriate.

**Suggestions:**

- Propose future studies using omics-based approaches, e.g., epigenetics, transcriptomics, to explore serotonergic dysregulation.
- Consider including a graphical summary of key findings and proposed mechanisms (i.e., a visual version of the expanded Triple Risk Model).

**Minor Editorial Notes**

- Consistency in abbreviation use (e.g., PostKCOD vs. post-KCOD) should be ensured.
- The manuscript is long; consider condensing the discussion slightly while maintaining its depth.
- Check for typographical errors in statistical tables (e.g., inconsistent decimal formatting or missing values).

**Final Summary**

The manuscript contributes valuable new insights into the neuropathology of SIDS, particularly in high-risk, socioeconomically vulnerable populations. The observed elevation of 5-HT1A receptor binding in premature SIDS cases challenges previous assumptions and suggests potential subtypes of serotonopathy. While the study is well-executed and rigorously presented, few clarifications, analyses, and interpretive expansions are needed before publication.
