# Supplementary material for: Serotonergic receptor binding in the brainstem in the Sudden Infant Death Syndrome in a high-risk population
Source: PLoS One. 2025 Sep 10;20(9):e0330940. doi: 10.1371/journal.pone.0330940 (PMC12422518; doi:10.1371/journal.pone.0330940)
Supplement: Attachment — Submitted filename: Response to Reviewers FINAL 71624 FINAL.docx [file pone.0330940.s006.docx]

In the following document, we respond to each of the concerns/suggestions of the reviewers according to your instructions.  The editor/reviewer comments are noted by either a number or a bullet point. Our response follows the comment.

1. We have ensured that our manuscript meets PLOS One’s style requirement.
2. Funding Statement. We have removed the funding statement from the manuscript and placed it in this cover letter as instructed (see below). We have also placed this statement in the cover letter as requested.

Revised Funding Statement:

The research reported in this publication was supported by National Institutes of Health (NIH) grants U01HD045935 (MEN, JA, AJE, LLN, BBR, MAS ), U01HD055155 (WPF), U01HD045991 (HCK, RDF, KGB, JRD, DSP), and U01AA016501 (LB, EHB, JAC, HJO) funded by the National Institute on Alcohol Abuse and Alcoholism (https://www.niaaa.nih.gov/), *Eunice Kennedy Shriver* National Institute of Child Health and Human Development (https://www.nichd.nih.gov/), and the National Institute on Deafness and Other Communication Disorders (<https://www.nidcd.nih.gov/>). There was no additional external funding received for this study.

The work was performed in the context of the research network Prenatal Alcohol, SIDS, and Stillbirth (PASS) Network. This Network included affiliates of the NIH who participated in the overall PASS study design, decision to publish, and preparation of the manuscript.

3. Data Availability Statement

We have revised our data sharing statement (see below). Please note that we are unable to share data from tribal participants. There were 3 infants in our cohort from tribal communities in the Northern Plains.

Revised Statement: De-identified data from the Safe Passage Study is available through NICHD’s Data and Specimen Hub ([DASH](https://urldefense.com/v3/__https:/secure-web.cisco.com/14plsk4aXRTTOjQgzWr29wUG6uTCIDCGSOEKGAOWUaKSLDXjtr9qzPj5hEzpZwmHNneNW2eeON6soC82OtBAL321Wsl8ARSQ-x-C2MZTeeGZrTHwC4zNKMAjCOQRK3VKmoqrm9o8LDLkz9PkLBZ7gW4njKYlXOJqJymS78Hc8kzsb4XpVfVz72ef0k0pGNBZppgxocu9lNfvcZ5gfZ_kIczAJXLhdSR_C7TOD1iu_Jqp-YrJjIwsCEMsW3fyYzANma2haEqPV3x4K2wTypGeSvLBHWJhPdo1xwtvMC8oov52oxk1Kw1NCJbzySXEWp_7yj-5sxASnkbEjwhoi5jF2Eg/https*3A*2F*2Furldefense.com*2Fv3*2F__https*3A*2Fdash.nichd.nih.gov*2F__*3B*21*21NZvER7FxgEiBAiR_*21vLi3M1CpbSUecsa1_oorxcDDKyFZ8lc8_SVsilLebBaEIlry_tmEUCQxgjFuSIbcPTu3fihPPgQfpx40ER29IQ7JNSYK1L_HrWJkKQXv*24__;JSUlJSUlJSUlJSUlJQ!!NZvER7FxgEiBAiR_!shjUCmZ8SmYMiAHVpN8XQzMIOMrr0q8Wg1E0f4pIR6vm_o3PApEsbM0QcIhW4xgOHROzPdilmVw4vK6UcyOQrYgVx5Y0SJdZDY6DBseQ$)). All case demographic and exposure data are available on DASH. Elliott, Amy (2025). A Prospective Study on the Role of Prenatal Alcohol Exposure in SIDS and Stillbirth (Version 1). NICHD Data and Specimen Hub.  <https://doi.org/10.57982/sv8c-4y07>. Ligand receptor binding data are available as Supplemental File 2. In compliance with tribal data sharing agreements, we have excluded 3 cases from American Indian tribes in the Northern Plains from these publicly shared datasets.

4. The instructions asked to amend the manuscript submission data (via Edit Submission) to include author Hannah C Kinney MD. We are unsure what is needed, since Hannah Kinney is listed as the first author under “Current Author List”

## We have provided all of the data related to sleeping conditions in the text under “Clinicopathological Information”. We removed the “Data Not Shown” statement.

5. We have provided captions for our supporting information files at the end of the manuscript.

Reviewer #1.

- Clarify the timing for the public release of receptor binding data.

-The receptor binding data is now provided as a supplemental file (see the Data Availability Statement above)

- Explicitly state in the main manuscript whether any outliers or missing data were excluded and how.

-We have added the following statement to the statistical methods. “There were no outliers excluded from statistical analysis and complete case analysis was performed.”

- More explicitly address why some expected correlations (e.g., with maternal smoking or alcohol use) were not statistically significant, despite high exposure prevalence.

-We have added the following statement to the limitations section.

“One of our original hypotheses was regarding the effect of prenatal exposures on the serotonergic system, specifically, 5-HT_1A_ binding in SIDS.   However, a second limitation to the study was the fact that all mothers (SIDS and controls) smoked during pregnancy, and many drank alcohol.  Statistically we saw no effect of drinks per pregnancy or number of cigarettes per week on 5-HT_1A_ receptor binding. Although this lack of effect may suggest that alcohol and smoking have limited effects on binding, it may also reflect an inability to meaningfully parse out any relationship between exposure and binding, due to the near-ubiquitous use of cigarettes and alcohol “

- Reviewer #3. “While the study is well-executed and rigorously presented, few clarifications, analyses, and interpretive expansions are needed before publication.”

-We have attempted to identify and clarify the study statements where noted by the reviewers.

- Reviewer #4. “(The manuscript) requires stronger justification and more transparent reporting, especially in relation to multiple comparisons and the modeling of small subgroups.”

-We have added the following statement at the end of the Statistical Methods section. “In all analyses, a p-value≤0.05 was accepted for statistical significance. No formal adjustment for multiple testing was performed, but consistency of results across multiple related outcomes is emphasized.”

-We acknowledge that are sample sizes are relatively small and have noted this in the limitations. Our overall case numbers in this study, however, are in line with other SIDS autopsy studies published by us and others. The premature subsets of SIDS and controls are larger than any other SIDS autopsy study to date which allowed for the analyses of prematurity−−analyses considered to be exploratory in nature and warranting replication in larger datasets.

- “The interpretation of exposure-related findings should be more cautious, as the cohort’s near-universal exposure to smoking and alcohol limits the ability to draw meaningful conclusions.

-We agree that we cannot draw meaningful conclusions about the effect of exposure. We have emphasized this point in the limitations section (see comments to Reviewer #1).

- “Additionally, some claims made in the discussion overstate the generalizability of the findings and should be revised for a more measured tone.”

-We have taken a more measured tone on certain statements in the discussion. We have also included the following statement: “While the results of the study are readily generalizable to high-risk population with prenatal smoking and alcohol intake and prematurity, the results suggest that pre- and/or postnatal stress is the common denominator and reflects the burden and type of stress in each population, with the overall nature and burden of the stress being unique in each population. “

Other Peer Review comments: #1.

- Regarding the mention of maternal anxiety and depression in the abstract, “Please, kindly consider specifying p-values or clarifying the statistical significance of these findings”.

-In order to make suggested additions to the abstract while maintaining the necessary word count, we took out the sentence regarding anxiety and depression. We added p-values for other SES proxies.

- “[The introduction] introduces “serotonopathies” appropriately but could briefly clarify the mechanistic importance for non-specialist readers”

-We have added details of the mechanistic importance of serotonin in the 2^nd^ paragraph of the introduction as suggested.

- The text mentions “triple-risk model” and intrinsic vulnerability but doesn’t explain how this informs the sampling or statistical approach. Briefly aligning the cohort selection with this model.

-The Triple risk model is a model or hypothesis of the pathogenesis of SIDS. It does not inform the sampling or statistical approach. This was an autopsy study and all available cases and controls were included in the study if technically usable. Among the cohort available to us for analysis, the three components of the model reflected characteristics of the unique study population and drove the interpretation of our results.

- The rationale for grouping SIDS deaths based on postconceptional age is justified but consider explaining how gestational age was estimated (e.g., clinical records, last menstrual period).

-Gestational age at enrollment was determined during the first prenatal visit using standard clinical practices at each study center – ultrasound in South Africa, and a combination of clinical examination, ultrasound, and last menstrual period in the Northern Plains. This was previously reported in Dukes et al Alcohol. 2017 August; 62: 49–60. This has been added to the text.

- It's unclear if any matching strategy (for postconceptional age) was employed when selecting controls.

-There was no matching of cases with controls. This was an autopsy study, and all available cases were included.

- Although it's noted that cases were excluded if the pons were damaged, it would help to provide the number or percentage of cases excluded for transparency and potential selection bias assessment.

-We have added the following information into the Clinical Database part of the manuscript. “Of the 14 SIDS and 10 PostKCOD controls available and of suitable quality for autoradiography, only 9 (64%) SIDS had rostral pons available but with varying availability of regions of interest within the pons (n=6-9). Four PostKCOD controls (40%) had rostral pons available.

- There is no mention of whether assay batch effects were controlled for (e.g., all samples run in a single batch or randomized across batches).

We added the following statement to the Methods section: “Autoradiography binding was performed in batches with cases blinded to diagnosis and randomly distributed across the different batches. The same autoradiography standard was used across the different batches.”

- Missing data on exposures were imputed as “not exposed” or “not diagnosed.” This assumption is highly problematic and may introduce systematic misclassification bias. If such an assumption was necessary, a sensitivity analysis excluding imputed cases should be performed and results compared

-The method for the imputation is published in multiple studies, cited below. These manuscripts have been added to the text.

Sania A, Pini N, Nelson ME, Myers MM, Shuffrey LC, Lucchini M, Elliott AJ, Odendaal HJ, Fifer WP. K-nearest neighbor algorithm for imputing missing longitudinal prenatal alcohol data. Adv Drug Alcohol Res. 2025 Jan 28;4:13449. doi: 10.3389/adar.2024.13449. PMID: 39935524; PMCID: PMC11811783..

Shuffrey LC, Myers MM, Isler JR, et al; PASS Network. Association between prenatal exposure to alcohol and tobacco and neonatal brain activity: results from the Safe Passage Study. JAMA Netw Open. 2020;3(5):e204714. doi:10.1001/jamanetworkopen.2020.4714

- Testing interactions with PCA despite small sample sizes should be further justified. The authors acknowledge this, but an explanation of why they proceeded (such as effect sizes in previous studies) would strengthen transparency.

-Our analyses were planned *a priori* and interactions were tested based on previous published work that noted that the effect of age could differ by groups (See references).

Duncan JR, Paterson DS, Hoffman JM, Mokler DJ, Borenstein NS, Belliveau RA, et al. Brainstem serotonergic deficiency in sudden infant death syndrome. JAMA. 2010;303:430-7.

Haynes RL, Trachtenberg F, Darnall R, Haas EA, Goldstein RD, Mena OJ, et al. Altered 5-HT2A/C receptor binding in the medulla oblongata in the sudden infant death syndrome (SIDS): Part I. Tissue-based evidence for serotonin receptor signaling abnormalities in cardiorespiratory- and arousal-related circuits. J Neuropathol Exp Neurol. 2023;82(6):467-82

- Was any **multiple comparisons correction** (e.g., Bonferroni or FDR) considered given the number of nuclei analyzed?

-Due to the small number of cases, correction for multiple comparisons was not done. Although sample sizes were not amenable to warrant multiple testing corrections, our findings are strengthened by the consistency of results across several nuclei.

- The use of multiple linear regressions for each nucleus increases Type I error risk. Was any correction for multiple testing applied (Bonferroni or FDR)? If not, the risk of false positives should be explicitly discussed in the limitations.

-See the response above.

- k-Nearest Neighbor (kNN) imputation for missing binding values is acceptable, but the number of neighbors (k), the distance metric, and whether cross-validation was used to tune k are not described.
- While it's good to see kNN imputation used, more detail is needed: What was the rationale for choosing kNN over other methods (such as multiple imputation)?
- It's unclear which variables were used in the imputation model, and whether missingness was assumed to be Missing at Random (MAR), Missing Completely at Random (MCAR), or otherwise and whether any sensitivity analysis was done. A brief discussion of imputation assumptions would be useful.

-Please see the response above regarding kNN imputation. This method has been published.

- Given high exposure prevalence to alcohol and smoking in both SIDS and control groups, the authors correctly note that comparisons are underpowered. However, this severely limits conclusions related to prenatal exposures. Recommend emphasizing this limitation earlier in the abstract and results

-We had emphasized this in the original abstract and did not make changes. We have now emphasized this limitation in the Results and more fully describe it in the Limitations section of the Discussion.

- Interaction terms are tested but need clearer interpretation. Where significant diagnosis x age interactions are found, authors should clarify whether slopes differ by group or whether there is a crossover effect.

Our data with significant interaction effects suggests that SIDS is associated with higher binding compared to PostKCOD controls, but only at younger ages and only in preemies. We have added this point to the results section.

- Table 8 shows no significant effect of smoking or alcohol exposure on binding. This contradicts earlier hypothesis and highlights cohort limitations (ubiquitous exposure). Authors acknowledge this, but it should be reiterated in the discussion as a major constraint.

-We have highlighted this in the beginning of the Discussion and in the Limitations section, the latter with additional text added to emphasize this important point.

- Discussed epigenetic mechanisms are speculative and should be clearly stated as hypotheses without overstating evidence

-We have modified the text concerning epigenetics to clarify that it is a hypothesis.

- Claim that “serotonergic circuits are altered in all SIDS cohorts” may be too broad. Suggest qualifying this to reflect the study’s limitations and absence of a truly “healthy” control group.

-We have modified text from the discussion to now read: “While the SPS cohort has limitations (i.e, small numbers, lack of healthy controls, etc), as does any autopsy study, an important point is that medullary serotonergic circuits are altered in all SIDS cohorts that we have studied to date, irrespective of global background.”

Other Peer Review comments: #2.

- Include in the abstract a sentence highlighting the novel finding of increased 5-HT1A binding in premature SIDS infants.

-We have added this to the abstract

- Clarify early that the study was unable to test the influence of maternal smoking/drinking due to high prevalence in both groups.

-This limitation is noted in the abstract. We have attempted to emphasize it more in the Results and Discussion.

- Consider emphasizing earlier into the introduction, that prior studies observed *reduced* 5-HT1A binding in SIDS to better contrast the present findings.

-This point was previously made in the second paragraph of the Introduction with references to the manuscripts showing this reduction. To maintain the flow of information within the Introduction, we did not move or alter this section.

- Add a brief explanation (maybe a paragraph) of 5-HT1A receptor function in early brain development to frame the broader physiological implications.

## -We have added a short summary of the role in 5-HT1A to the section “Potential Consequences in Infant Physiology of a Medullary Serotonopathy in the SPS” in the Discussion.

- Provide a power analysis or justification for the sample size given the number of comparisons.

-Power analyses were done prior to the initiation of the PASS study in 2007. These are described in Dukes et al. The Safe Passage Study: Design, Methods, Recruitment, and Follow-Up Approach. Paediatr Perinat Epidemiol. 2014 September; 28(5): 455–465.

- Acknowledge the control group limitations more clearly (e.g., KCOD infants are not necessarily "healthy" controls).

## -We have modified the text related to controls as suggested in the “Limitations, Strengths, and Caveats”

- Clarify whether blinding was employed during receptor quantification.

-All analysis of receptor binding was done blinded to diagnosis and age. This has been added to the Methods section.

- Consider discussing whether post-mortem interval was controlled beyond the statistical statement that it had no effect.

-The lack of PMI effect on the data was determined by linear regression. We have added this to the Statistical Analysis section.

- Suggestions for Complementary Approaches:

-The reviewer suggests a number of excellent experiments to further our understanding of the data presented in this publication. We appreciate these thoughtful suggestions and agree they should be done. We are constrained by limited tissue for all of these markers and methods. They are part of planned future studies in the remaining precious samples. We appreciate that they are suggestions as they were beyond the scope of the present study.

- Highlight that inclusion of pre-discharge controls (PreKCOD) strengthens the interpretation of age-related trends.

-We have added to previous text to include the comment above. The text now reads: “While we acknowledge that the PreKCOD infants had serious health complications (Table 1), their inclusion strengthens the interpretation of age-related findings”

- Provide effect sizes and confidence intervals for binding comparisons to emphasize magnitude.

We thank the reviewer for this suggestion but have chosen not to provide effect sizes due to the inclusion of interaction terms in the modelling, as the effect size can vary by age.

- Consider modeling developmental trajectories of 5-HT1A binding using nonlinear regressions (e.g., splines).

We agree that this is an interesting statistical approach. Given the small numbers we feel the sample size may be inadequate for meaningful nonlinear modeling. We did not make changes regarding this comment.

- Sex-specific analysis: Are there sex differences in receptor binding or SIDS incidence in this cohort?

-We agree that this is an interesting question, particularly given the increased risk of SIDS in males. In the PASS cohort presented, we did not, however, test for sex differences. Given the sample sizes, these analyses would likely not provide meaningful data. In addition, our other studies of serotonin-related abnormalities in SIDS did not uncover an effect of sex on the data.

- Cause-of-death heterogeneity in controls: Address whether the control group includes heterogeneous mechanisms of death that could confound comparisons.

## -A note about the heterogenous causes of death has been added to the Results (Clinico- pathological Information) and the Limitations section of the Discussion.

- Expand on potential mechanisms of elevated 5-HT1A binding (e.g., stress-induced overexpression, altered feedback signaling).

-We had included in the original submission the following statement: “We may speculate that increased 5-HT_1A_ binding may reflect increased receptor numbers, which could be related to impaired dendritic pruning and increased numbers of dendritic spines – all possible responses to stress. While not fully resolved, the mechanisms behind these developmental effects are likely multifactorial, involving elevated levels of cortisol and other stress hormones, intricate fetal-placental-maternal interactions, reprogramming of vulnerable fetal neural networks, and possibly epigenetic effects.” Beyond this speculation animal models are necessary to address mechanistically what underlies the pathological findings. Given the length of the article already, we did not speculate beyond what is written.

- Discuss possible functional implications: does increased 5-HT1A binding imply increased inhibitory tone in cardiorespiratory centers?

## -We had written in the original submission a brief summary of potential functional implications of increased 5-HT1A binding in different nuclei of the medulla. This is written in “Potential Consequences in Infant Physiology of a Medullary Serotonopathy in the SPS”. We have expanded this section to better address the normal role of 5-HT1A binding to better frame the potential consequences noted.

- Briefly explore how this subset of "preterm SIDS" may represent a distinct pathophysiological endotype.

-We added the following to the Conclusions section. “In addition to high 5-HT_1A_ binding in SIDS infants, a unique, unexpected feature of the current study was the high proportion of infants born prematurely, which drove our main findings, and may have uncovered a unique, and not previously well appreciated, subset or pathophysiological endotype of SIDS in premature infants with serotonin abnormalities distinct from SIDS infants born at term.”

- Consider referencing animal studies of maternal stress or early-life adversity affecting serotonergic signaling.

## -Animal studies related to this topic are provided under the header “Maternal Stress Related to Socioeconomic Conditions: Impacts on the Development of the 5-HT System and SIDS Risk”

- Note the lack of external validation (e.g., protein expression or imaging).

## -We have added a statement about this in the Limitations, Strengths, and Caveats section of the manuscript.

- Discuss the potential influence of systemic infections in controls on receptor binding.

-The potential of systemic infection influencing receptor binding is an important question and we appreciate the reviewer bringing attention to it. The prevalence of systemic infection in the Safe Passage Study control cohort is higher than our previously published San Diego control cohorts. We have examined carefully the 5-HT_1A_ receptor binding across the controls of both populations and have found no consistent differences. This supports the conclusion that infection did not significantly alter binding in the SPS study controls.

- Propose future studies using omics-based approaches, e.g., epigenetics, transcriptomics, to explore serotonergic dysregulation.

## -We have added a statement about this in the Limitations, Strengths, and Caveats section of the manuscript.

- Consider including a graphical summary of key findings and proposed mechanisms (i.e., a visual version of the expanded Triple Risk Model).

-This suggested model was given in the original manuscript. See Figure 5. “Proposed expanded model of the pathogenesis of a medullary serotonopathy in a high-risk population of SIDS.”

**Minor Editorial Notes**

- Consistency in abbreviation use (e.g., PostKCOD vs. post-KCOD) should be ensured.

-We have corrected inconsistency where we noted them.

- The manuscript is long; consider condensing the discussion slightly while maintaining its depth.

While we took steps to shorten the manuscript, the additions requested for resubmission added to the overall word length.

- Check for typographical errors in statistical tables (e.g., inconsistent decimal formatting or missing values).

We have corrected inconsistency where we noted them.
