# Supplementary material for: Serotonergic receptor binding in the brainstem in the Sudden Infant Death Syndrome in a high-risk population
Source: PLoS One. 2025 Sep 10;20(9):e0330940. doi: 10.1371/journal.pone.0330940 (PMC12422518; doi:10.1371/journal.pone.0330940)
Supplement: Attachment — Submitted filename: Peer Reviewer Comments SIDS.docx [file pone.0330940.s004.docx]

**Peer Reviewer Comments:** **Serotonergic Receptor Binding in the Brainstem in the Sudden Infant Death Syndrome in a High-Risk Population**

**Abstract**

- The abstract clearly outlines the hypothesis, study design, and major findings.
- Here, it was mentioned that a "trend for maternal anxiety and depression" in mothers of SIDS infants was present. Please, kindly consider specifying p-values or clarifying the statistical significance of these findings.

**Introduction**

- Provides a strong scientific rationale and integrates well-established prior work on serotoninergic abnormalities in SIDS.
- Here, it introduces “serotonopathies” appropriately but could briefly clarify the mechanistic importance for non-specialist readers.

**Materials and Methods**

**Study Design**

- Clear diagnostic criteria for SIDS, post-KCOD, and controls are defined, with references to prior studies and the San Diego SIDS Data.
- The text mentions “triple-risk model” and intrinsic vulnerability but doesn’t explain how this informs the sampling or statistical approach. Briefly aligning the cohort selection with this model
- The rationale for grouping SIDS deaths based on postconceptional age is justified but consider explaining how gestational age was estimated (e.g., clinical records, last menstrual period).
- It's unclear if any matching strategy (for postconceptional age) was employed when selecting controls.

**Tissue Preparation and Binding Assays**

- The receptor binding assay methodology is well described, referencing prior validated protocols.
- Tissue collection and handling procedures are outlined to ensure reproducibility and interpretability of the binding data.
- Although it's noted that cases were excluded if the pons were damaged, it would help to provide the number or percentage of cases excluded for transparency and potential selection bias assessment.
- There is no mention of whether assay batch effects were controlled for (e.g., all samples run in a single batch or randomized across batches).

**Exposure and Clinical Data Collection**

- The integration of maternal exposure history (smoking, and alcohol)
- Missing data on exposures were imputed as “not exposed” or “not diagnosed.” This assumption is highly problematic and may introduce systematic misclassification bias. If such an assumption was necessary, a sensitivity analysis excluding imputed cases should be performed and results compared.

**Statistical Analysis**

- The modeling strategy is sound: ANOVA, chi-square, and linear regression are used appropriately.
- Adjustment for postconceptional age (PCA) is correct and necessary, given known developmental effects.
- Testing interactions with PCA despite small sample sizes should be further justified. The authors acknowledge this, but an explanation of why they proceeded (such as effect sizes in previous studies) would strengthen transparency.
- Was any **multiple comparisons correction** (e.g., Bonferroni or FDR) considered given the number of nuclei analyzed?
- The use of multiple linear regressions for each nucleus increases Type I error risk. Was any correction for multiple testing applied (Bonferroni or FDR)? If not, the risk of false positives should be explicitly discussed in the limitations.
- k-Nearest Neighbor (kNN) imputation for missing binding values is acceptable, but the number of neighbors (k), the distance metric, and whether cross-validation was used to tune k are not described.
- While it's good to see kNN imputation used, more detail is needed: What was the rationale for choosing kNN over other methods (such as multiple imputation)?
- It's unclear which variables were used in the imputation model, and whether missingness was assumed to be Missing at Random (MAR), Missing Completely at Random (MCAR), or otherwise and whether any sensitivity analysis was done. A brief discussion of imputation assumptions would be useful.

**Results – Demographics and Exposure**

- Table 2 and Table 3 effectively summarize infant and maternal characteristics.
- Given high exposure prevalence to alcohol and smoking in both SIDS and control groups, the authors correctly note that comparisons are underpowered. However, this severely limits conclusions related to prenatal exposures. Recommend emphasizing this limitation earlier in the abstract and results.

**Results – 5-HT1A Binding**

Excellent description of binding across developmental stages. Use of PCA as a covariate is appropriate.

- Table 6 – Interaction terms are tested but need clearer interpretation. Where significant diagnosis x age interactions are found, authors should clarify whether slopes differ by group or whether there is a crossover effect.
- The inclusion of both PreKCOD and PostKCOD to reflect a developmental continuum is well justified.

**Exposure Effects on Binding**

- Table 8 shows no significant effect of smoking or alcohol exposure on binding. This contradicts earlier hypothesis and highlights cohort limitations (ubiquitous exposure). Authors acknowledge this, but it should be reiterated in the discussion as a major constraint.

**Discussion**

Thorough and conceptually strong. Effectively connects serotonin findings to stress, social determinants of health, and brain development.

- Discussed epigenetic mechanisms are speculative and should be clearly stated as hypotheses without overstating evidence.
- Claim that “serotonergic circuits are altered in all SIDS cohorts” may be too broad. Suggest qualifying this to reflect the study’s limitations and absence of a truly “healthy” control group.

**Overall Recommendation**: **Minor Revisions**

The manuscript addresses an important scientific question and is based on rich and valuable histopathological data. The statistical approach is fundamentally sound, particularly in its use of regression models and adjustments for postconceptional age; however, it requires stronger justification and more transparent reporting, especially in relation to multiple comparisons and the modeling of small subgroups. The interpretation of exposure-related findings should be more cautious, as the cohort’s near-universal exposure to smoking and alcohol limits the ability to draw meaningful conclusions. Additionally, some claims made in the discussion overstate the generalizability of the findings and should be revised for a more measured tone. With these revisions, the manuscript would be suitable for publication.
